# Supplementary material for: Overexpression of a Fragaria vesca 1R-MYB Transcription Factor Gene (FvMYB114) Increases Salt and Cold Tolerance in Arabidopsis thaliana
Source: Int J Mol Sci. 2023 Mar 9;24(6):5261. doi: 10.3390/ijms24065261 (PMC10048884; doi:10.3390/ijms24065261)
Supplement: Supplementary file 1 [file ijms-24-05261-s001.zip › ijms-2154882-supplementary.pdf]

Table S1. List of primers used in this study.

Primer Name	PrimerSequence (5′→3′)	Purpose
AtSOS1-F	CTGCTTGCTACATTTCTGC	qPCR
AtSOS1-R	TGCTTCCTCTCCTTCCTT	qPCR
AtSOS3-F	GAATCCATCGCTCATCAA	qPCR
AtSOS3-R	CCATTTCTTCCTCTTCACA	qPCR
AtNHX1-F	AGCCTTCAGGGAACCACAAT	qPCR
AtNHX1-R	CTCCAAAGACGGGTCGCATG	qPCR
AtLEA3-F	GATTGACCCGGCTGAGCTACGA	qPCR
AtLEA3-R	AGATGGGATTCACCACAAAAGA	qPCR
AtCCA1-F	TTTGAGGCTTTATGGTAG	qPCR
AtCCA1-R	TTTCTGAGCGTGACTTCT	qPCR
AtCOR4-F	GAATCACCAGCGACGACA	qPCR
AtCOR4-R	CCCCAAGAAATCAAACAA	qPCR
AtCBF1-F	CCGACTTGTTGGATAATATGGCTGAAG	qPCR
AtCBF1-R	TTAGTAACTCCAAAGCGACACGTCACC	qPCR
AtCBF3-F	TTTACACGGCGGAACAGA	qPCR
AtCBF3-R	CACTGTACGGACGGAAGC	qPCR
FvActin-F	GCGACAATGGAACTGGAATGG	qPCR
FvActin-R	GACAATTTCCCGTTCAGCAGTG	qPCR
FvMYB114-F	ATGGAGGAGCAGACGGGT	full-length cDNA of FvMYB114
FvMYB114-R	CTATTCCGTATTATGCAAGATTTGTG	full-length cDNA of FvMYB114
HR-F	AGAACACGGGGGACGAGCTCATGGAGGAGCAGACGGGT	PCR for homologous recombination
HR-R	ACCATGGTGTCGACTCTAGACTATTCCGTATTATGCAAGATTTGT	PCR for homologous recombination
FvMYB114-qF	GGAGGAGCAGACGGGTTT	qPCR
FvMYB114-qR	TTGCCTAAGAGTTTGTGA	qPCR
FvMYB114-slF	GTCGACATGGAGGAGCAGACGGGT	For subcellular localization
FvMYB114-slR	TTCCGTATTATGCAAGATTTGTGGGATCC	For subcellular localization

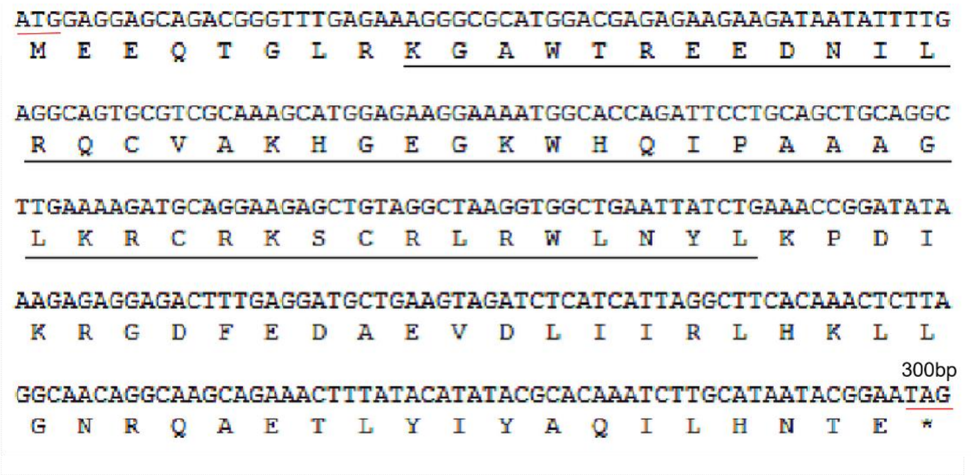

Figure S1. Sequence of *FvMYB114*.. The whole length of *FvMYB114* is 300bp, encoding 99aa. Underline means conserved DNA-binding domain of *FvMYB114*. ∗ means termination codon.

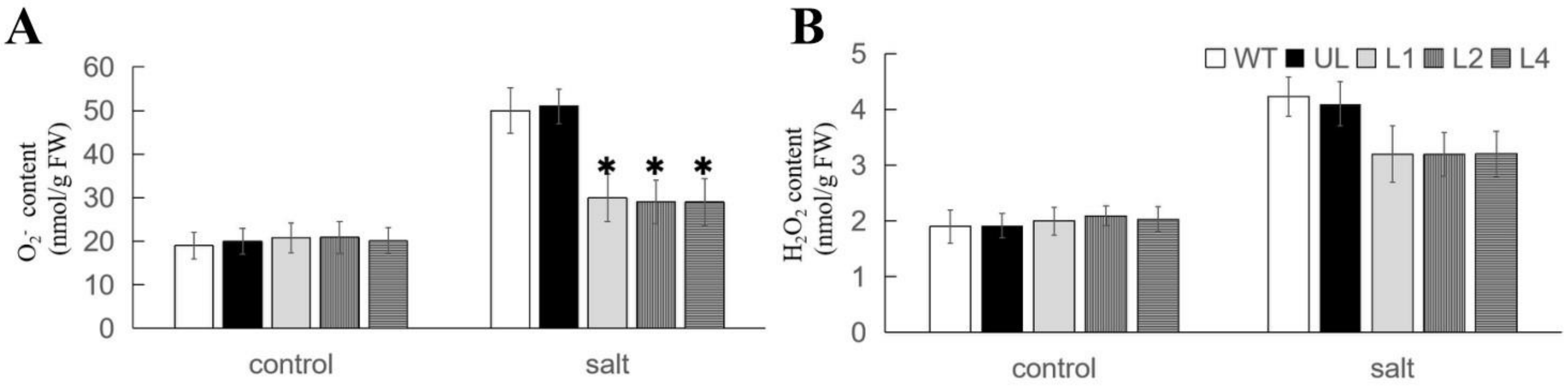

Figure S2. O_2^- content and H_2O_2 content were tested between WT , UL and transgenic *Arabidopsis* under salt treatment. (**A**) O_2^- content of WT, UL, L1, L2 and L4 under control and salt condition; (**B**) H_2O_2 content of WT, UL, L1, L2 and L4 under control and salt condition. * $P \leq 0.05$

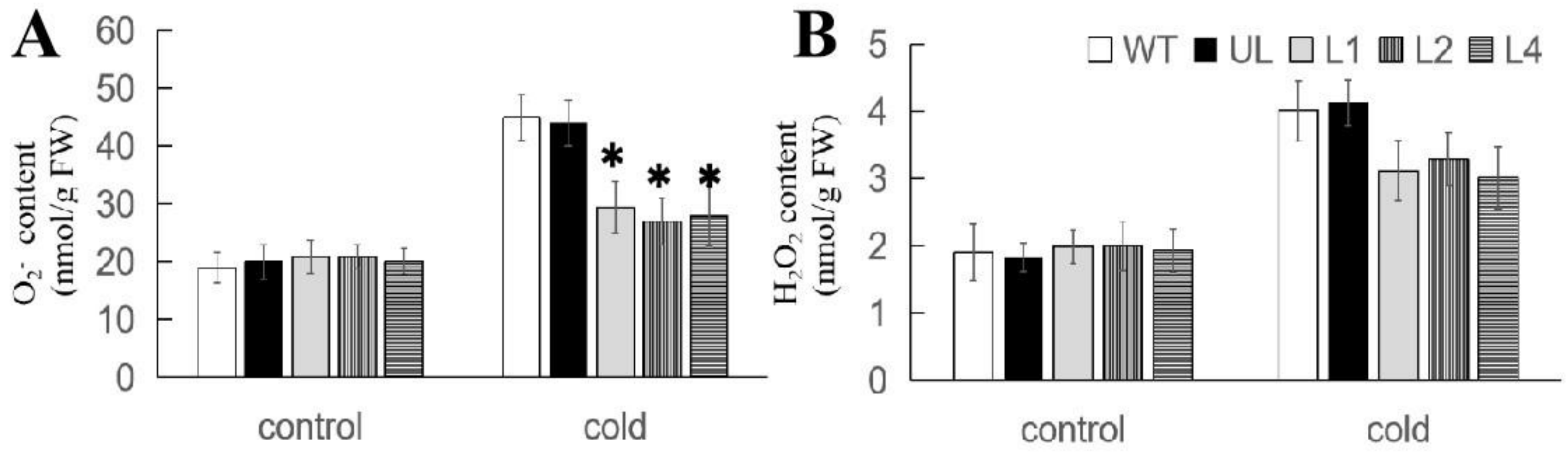

Figure S3. O_2^- content and H_2O_2 content were tested between WT , UL and transgenic *Arabidopsis* under cold treatment. (**A**) O_2^- content of WT, UL, L1, L2 and L4 under control and cold condition; (**B**) H_2O_2 content of WT, UL, L1, L2 and L4 under control and cold condition. * $P \leq 0.05$.
